# Sleeping green: an Italian survey for the assessment of the relationship between sleep and vegetarian diet

**DOI:** 10.1007/s11325-026-03593-3

**Published:** 2026-03-10

**Authors:** Maurizio Gorgoni, Alessio Comparelli, Sofia Frappetta, Valentina Alfonsi, Ludovica Annarumma, Elisa Pellegrini, Milena Camaioni, Alessandro Couyoumdjian, Serena Scarpelli, Luigi De Gennaro

**Affiliations:** 1 Department of Psychology, Sapienza University of Rome, Via dei Marsi 78, Rome, 00185 Italy; 2 Body and Action Lab, IRCCS Fondazione Santa Lucia, Rome, Italy; 3 Departmental Faculty of Medicine and Surgery, UniCamillus-Saint Camillus International University of Health Sciences, Rome, Italy

**Keywords:** Vegetarian, Vegan, Omnivore, Sleep, OSAS, Hypnic jerks

## Abstract

**Purpose:**

Plant-based diets are beneficial for health and sleep. Nevertheless, results on the relationship between entirely vegetarian (veg) diets and sleep are scarce and heterogeneous, and many relevant variables are rarely considered. We hypothesize that veg diets may be differently related to several sleep measures considering the role of other intervening factors.

**Methods:**

We used an online survey to collect self-reported data about dietary patterns, sleep quality, insomnia symptoms, sleepiness, obstructive sleep apnoea (OSA) risk, and sleep-related movement disorders. Socidemographic variables, adherence to the Mediterranean diet, mental health, eating disorder symptomatology, and chronotype were also considered.

**Results:**

Our final sample included 747 participants: 532 omnivores (omniv) and 215 veg. Compared to ominv, veg exhibited a lower risk of OSA, a higher frequency of hypnic jerks, and a lower tendency to eveningness, without differences in sleep quality, insomnia symptoms, sleepiness, and other sleep-related movement disorders. Multiple regression models showed that considering the role of other variables the dietary pattern only predicted OSA risk (i.e., greater OSA risk in omniv) and hypnic jerks (i.e., higher hypnic jerk frequency in veg).

**Conclusion:**

Our findings suggest that an utterly veg diet can affect several sleep variables differently, mainly reducing the risk of OSA and increasing the frequency of hypnic jerks. We highlight the relevance of a thorough assessment of sleep measures associated with the veg diet and the importance of controlling for other confounding factors to reach a more nuanced understanding of the relationship between dietary patterns and sleep.

**Supplementary Information:**

The online version contains supplementary material available at 10.1007/s11325-026-03593-3.

## Introduction

Although the benefits of plant-based diets on sleep are well-documented [1], the effects of entirely vegetarian (veg) diets compared to omnivore (omniv) ones are less studied. Veg diets are not equally healthy: restrictive diets characterized by poor presence of specific nutrients or diets rich in highly processed and refined food are associated with increased morbidity and mortality [2]. Furthermore, it is unclear whether the advantage of healthy veg diets represents an all-or-nothing phenomenon and whether mainly plant-based diets with low amounts of animal products, like the Mediterranean diet, have positive, negative, or neutral effects on health [2].

Several findings suggest longer sleep duration [3, 4] and reduced insomnia risk [5] and symptoms [6] in veg. In an intervention study, participants who followed veg diet for three months reported better sleep quality than those on non-veg diet [7]. Omniv exhibited longer light sleep while veg were more refreshed upon waking up during a one-week assessment [8]. However, the direct comparison between veg, pescetarians, and omniv did not show differences in sleep quality [9]. No relationship was found between type of diet and sleep quality in female college students [10], and no differences in insomnia symptomatology have been observed between veg and omniv in Lebanon [11]. Finally, veg reported better sleep quality compared to omniv but such difference disappeared after adjusted for depression [12].

Overall, the literature about sleep in veg diets is small and heterogeneous. Findings on sleep quality are conflicting and conducted with different methods. Sleep variables other than quality and duration are not frequently considered. One of the main predictors of obstructive sleep apnoea (OSA) is obesity [13], and weight loss have a beneficial effect on patients with OSA [14]. Nevertheless, the association between completely veg diets and OSA risk is understudied.

While veg are more likely to exhibit iron deficiency compared to non-veg [15] and low levels of serum ferritin characterized many sleep-related movement disorders [16], no information is currently available on the potential relationship between veg diets and sleep-related movement disorder.

Many studies about the relationship between sleep features and dietary patterns do not consider the possible role of other factors. Beyond the relevance of sociodemographic variables, the role of mental health should be better investigated. Given that individuals with eating disorders are characterized by poor sleep quality [17], and several studies point to an association between eating disorders and veg diets [18], the influence of eating disorders on the relationship between sleep and dietary patterns should be considered. Finally, chronotype has been substantially ignored in this field, although greater eveningness seems associated with a higher engagement with unhealthy dietary habits [19].

We hypothesize that veg diets are differently related with several sleep measures, considering the role of other intervening factors. The aim of the present study is to assess the relationship between veg diets and sleep in an Italian sample, assessing self-reported sleep quality, insomnia symptoms, sleepiness, OSA risk, and sleep-related movement disorders, also considering the role of socidemographic variables, adherence to the Mediterranean diet, mental health, eating disorders, and chronotype.

## Methods and materials

### Design and participants

We collected data in an Italian sample using an anonymous online survey in Google Forms shared on social media and through a flyer with a QR code distributed in the Rome metropolitan area. Participants had access to the survey after reading the informed consent form, declaring explicit agreement to participate in the research. We included only participants with age ≥ 18 years.

The study was approved by the Institutional Review Board of the Department of Psychology of the Sapienza University of Rome (#0002284/2022) and conducted in accordance with the Declaration of Helsinki.

Our final sample included 747 participants: 532 omniv, 118 vegans, and 97 vegetarians. Vegan and vegetarians were pooled together in a veg group of 215 participants after controlling for the absence of significant differences in sleep variables (Table S1). Excluded cases and diet durations are also reported in the Supplementary Material.

### Materials

- Age, gender, physical activity, presence of illness, smoking, alcohol, weight and height (to assess Body Mass Index – BMI) were collected.
- Diet: we used a single question to investigate the participants’ diet (i.e., omnivore, vegetarian, vegan, pescetarian).
- Mediterranean Diet Adherence Screener (MEDAS) [20] is a 14-item questionnaire to assess adherence to the Mediterranean diet. The global score ranged from 0 to 14, with higher scores indicating greater adherence to the Mediterranean diet.
- Eating Disorder Examination Questionnaire (EDE-Q) [21] is a 28-item questionnaire for assessing eating disorder psychopathology and behaviours. Higher scores reflect greater severity/frequency of a behaviour. Items are grouped into four subscales reflecting several psychopathological features of eating disorders. The global score is represented by the mean of the four subscales.
- Depression Anxiety Stress Scales (DASS-21) [22] is a questionnaire in which participants are asked to rate frequency and severity of depression, anxiety, and stress symptoms. Higher scores index greater symptoms.
- World Health Organization-Five Well-being Index (WHO-5) [23] consists of five items (0–5 scale) measuring the overall psychological wellbeing. A higher score reflects a higher quality of life.
- Pittsburgh Sleep Quality Index (PSQI) [24] is a 19-item questionnaire to investigate sleep quality, resulting in seven sub-scales and a sleep quality global score. A PSQI global score > 5 indicates poor sleep quality.
- Insomnia Severity Index (ISI) [25] is a 7-item (0–4 scale) questionnaire to assess the perceived severity of insomnia symptoms. A total score < 8 points to no insomnia, 8–14 subthreshold clinical insomnia, 15–21 insomnia of moderate severity, and > 21 severe insomnia.
- Epworth Sleepiness Scale (ESS) [26] is a questionnaire to investigate subjective sleepiness. The individuals are asked to rate their habitual chances of dozing off or falling asleep in 8 situations/activities. The score ranges from 0 (not sleepy et al.l) to 24 (extremely sleepy).
- Reduced Morningness-Eveningness Questionnaire (rMEQ) [27] is a 5-item questionnaire for assessing the individual chronotype. The score ranges from 4 to 25, with higher scores indicating a morning preference. We divided participants into evening-type, neutral-type, and morning-type according to the cut-off criteria.
- STOP-Bang [28] is a questionnaire assessing OSA risk. It consists of 8 “yes or no” questions on four subjective items and four demographic items. The final score ranges from 0 to 8; a score ≥ 3 is indicative of OSA.
- Munich Parasomnia Questionnaire (MUPS) [29] is a 22-item questionnaire assessing the presence and frequency of altered nocturnal behaviors on a Likert scale from 1 (“Never”) to 7 (“Very frequently-every or nearly every night”). For the present study, we considered the following altered motor nocturnal behaviors: hypnic jerks, rhythmic foot tremors, rhythmic movement disorder, periodic leg movements, and nocturnal leg cramps.

### Statistical analyses

Statistical analyses were performed using Jamovi 2.3.28. Chi-squared was used to assess the distribution of categorical variables in omniv and veg. Age, BMI, MEDAS, psychological variables, circadian preference, and sleep-related measures were compared between omniv and veg by unpaired Student’s t-tests. PSQI components and MUPS sleep-related movement disorders were compared between omniv and veg by Mann-Whitney U.

Multiple linear regressions were performed to assess the best predictor of sleep measures. In each model, one sleep-related measure was considered as the dependent variable, and the following variables were considered as predictors: diet, age, gender, BMI, physical activity, illness, smoke, alcohol, EDE, DASS-21, WHO-5, MEDAS, rMEQ. Multiple ordinal regressions with the same predictors were used when MUPS variables were considered dependent variables. Collinearity was controlled using a normal linear regression collinearity diagnostic model; no variance inflation factor ≥ 5 was observed.

## Results

### Sociodemographic characteristics

Considering sociodemographic features (Table 1), although both groups were predominantly composed of females, their proportion was significantly lower in the omniv group (χ^2^ = 7.01; *p* = 0.008). Compared to omniv, veg exhibited a significantly older age (t=-4.04; *p* < 0.001), higher adherence to the Mediterranean diet (t=-11.19; *p* < 0.001), lower BMI (t = 3.11; *p* < 0.001), greater proportion of participant with physical activity habits (χ^2^ = 14.4; *p* < 0.001), and a lower proportion of smokers (χ^2^ = 4.88; *p* = 0.03).

**Table 1:** Descriptive features of the total sample, the omnivore (omniv) and vegan/vegetarian (veg) groups. Distribution for dichotomous variables was assessed using the Chi-squared. Continuous variables were compared between groups using unpaired Student’s t-test. The alpha level was set at 0.05. The asterisk indexes a significant difference

	Total sample (*N* = 747)	Omniv (*n* = 532)	Veg (*n* = 215)	Omniv vs. Veg
				χ^2^ (*p*)
Variables				
Gender (M/F)	551/196	154/378	42/173	7.01 (0.008)*
Physical activity (Yes/No)	566/181	383/149	183/32	14.4 (< 0.001)*
Illness (Yes/No)	215/532	387/145	145/70	2.10 (0.15)
Smoke (Yes/No)	202/545	156/376	46/169	4.88 (0.03)*
Alcohol				
*Never* *Once a month or less* *Two/four times a month* *Two/three times a week* *At least four times a week*	134 175 290 112 36	88 115 218 87 24	46 60 72 25 12	9.45 (0.051)
	**Mean (SD)**			***t*** **(*p*)**
Age (y)	36.4 (14.5)	35.03 (14.51)	39.73 (14.05)	-4.04 (< 0.001)*
BMI	23.2 (5.80)	23.6 (6.43)	22.2 (3.55)	3.11 (< 0.001)*
MEDAS^1^	7.03 (2.0)	6.54 (2.00)	8.23 (8.00)	-11.19 (< 0.001)*

^1^ Analysis performed on 518 omniv and 210 veg

### Psychological measures and circadian preference

Table 2 reports psychological measures and circadian preference. Omniv exhibited a non-significant trend of higher eating disorder symptoms presence (EDE global score) compared to veg (t = 1.95; *p* = 0.051). Considering the EDE subscales, omniv showed significantly greater weight concerns compared to veg (t = 2.29; *p* = 0.02). Finally, veg exhibited a greater tendency to morningness compared to omniv (t=-3.57; *p* < 0.001).

**Table 2:** Results of EDE, DASS-21, WHO-5 and rMEQ questionnaires in the total sample and in omnivore (omniv) and vegan/vegetarian (veg) groups. The comparisons between groups have been performed using unpaired Student’s t-tests. The alpha level was set at 0.05. The asterisk indexes a significant difference

	Total sample (*N* = 747)	Omniv (*n* = 532)	Veg (n = 215)	Omniv vs. Veg
Variables	**Mean (SD)**			***t*** **(p)**
EDE Restrain	1.45 (1.52)	1.51 (1.53)	1.30 (1.47)	1.72 (0.09)
EDE Eating Concern	0.71 (1.09)	0.74 (1.08)	0.64 (1.10)	1.07 (0.29)
EDE Shape Concern	3.43 (2.30)	3.52 (2.32)	3.21 (2.24)	1.65 (0.10)
EDE Weight Concern	1.70 (1.56)	1.79 (1.57)	1.50 (1.50)	2.29 (0.02)*
EDE Global	1.82 (1.43)	1.89 (1.43)	1.66 (1.41)	1.95 (0.051)
DASS-21	17.7 (11.0)	17.82 (10.48)	17.45 (12.29)	0.42 (0.67)
WHO-5	13.9 (4.5)	13.83 (4.43)	13.90 (4.67)	-0.18 (0.85)
rMEQ	15.8 (3.88)	15.48 (3.91)	16.59 (3.71)	-3.57 (< 0.001)*

### Sleep and sleep-related variables

**Fig. 1:**
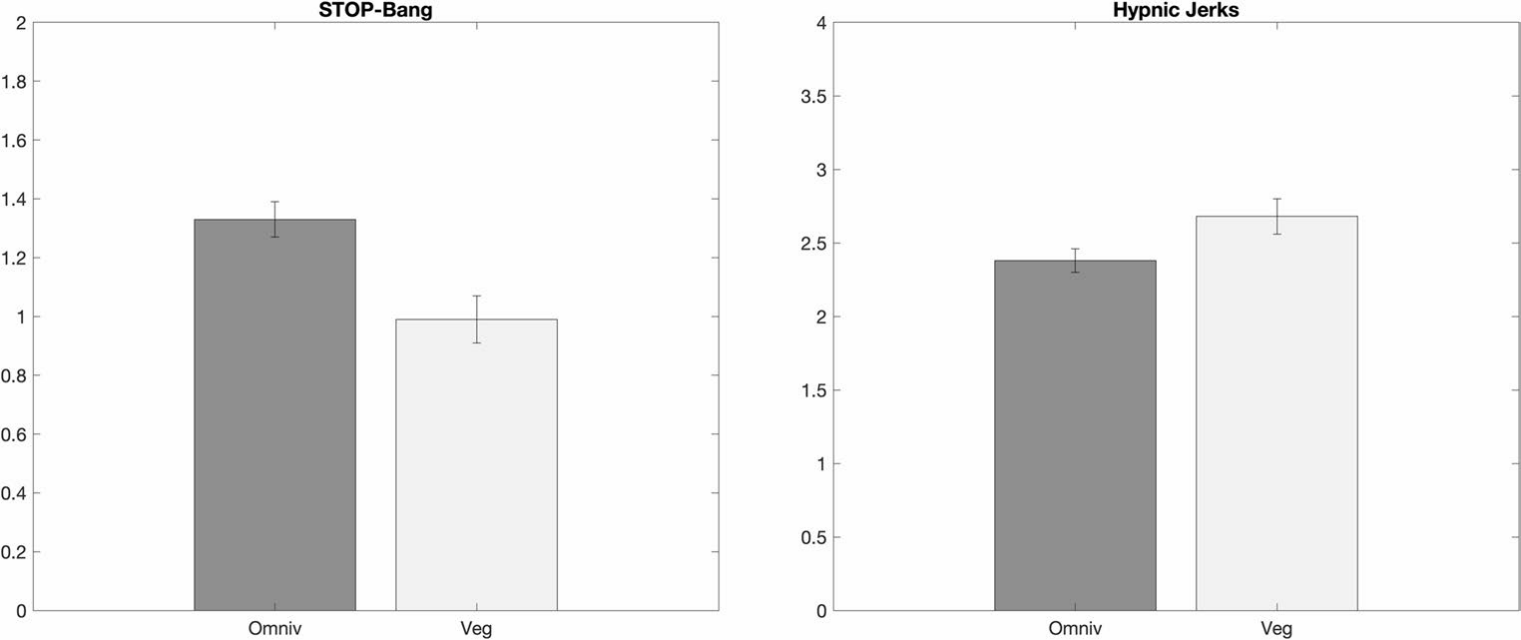
Significant results (*p* < 0.05) of the comparisons between omnivores (omniv) and vegan/vegetarians (veg) performed on the STOP-Bang (unpaired t-tests) and MUPS Hypnic jerks (Mann-Whitney U)

Concerning sleep and sleep-related measures (Table 3), veg exhibited a significantly lower risk of OSA (t = 3.37 *p* < 0.001) and more frequent hypnic jerks (*p* = 0.004) compared to omniv (Fig. 1).

**Table 3:** Sleep and sleep-related variables in the total sample and in omnivore (omniv) and vegan/vegetarian (veg) groups. The comparisons between groups have been performed using unpaired Student’s t-tests for PSQI Global, ISI, ESS, and STOP-Bang, and using Mann-Whitney U for PSQI components and MUPS sleep-related movement disorders. The alpha level was set at 0.05. The asterisk indexes a significant difference

	Total sample (*N* = 747)	Omniv (*n* = 532)	Veg (*n* = 215)	Omniv vs. Veg
Sleep and sleep-related variables	**Mean (SD)**			***t*** **(p)**
ESS	5.33 (3.55)	5.40 (3.55)	5.15 (3.55)	0.86 (0.39)
STOP-Bang	1.23 (1.27)	1.33 (1.32)	0.99 (1.125)	3.37 (< 0.001)*
ISI	6.17 (5.33)	6.10 (5.33)	6.37 (5.34)	-0.63 (0.53)
PSQI Global	5.95 (3.38)	5.93 (3.38)	6.0 (3.39)	-0.25 (0.80)
*PSQI Components*	**Mean (SD)**			***p***
C1 – Subjective sleep quality	1.11 (0.71)	1.11 (0.71)	1.11 (0.71)	0.97
C2 – Sleep latency	0.97 (0.97)	0.98 (0.98)	0.93 (0.93)	0.69
C3 – Sleep duration	0.98 (0.86)	0.97 (0.85)	1.02 (0.89)	0.49
C4 – Sleep efficiency	0.69 (0.95)	0.70 (0.95)	0.65 (0.95)	0.36
C5 – Sleep disturbance	1.06 (0.52)	1.04 (0.53)	1.08 (0.49)	0.35
C6 – Use of sleep medications	0.22 (0.70)	0.22 (0.71)	0.21 (0.66)	0.95
C7 – Daytime dysfunction	0.93 (0.70)	0.90 (0.66)	0.99 (0.76)	0.23
Sleep-related movement disorders	**Mean (SD)**			***p***
Hypnic Jerks	2.36 (1.82)	2.38 (1.82)	2.68 (1.78)	0.004*
Rhythmic Foot Tremors	1.30 (1.88)	1.26 (1.87)	1.40 (1.90)	0.37
Rhythmic Movement Disorder	0.44 (1.23)	0.44 (1.25)	0.42 (1.19)	0.99
Periodic Leg Movements	0.53 (1.19)	0.55 (1.23)	0.47 (1.11)	0.64
Nocturnal Leg Cramps	1.25 (1.55)	1.24 (1.56)	1.26 (1.52)	0.52

### Predictors of sleep and sleep-related variables

Table 4 reports the results of the multiple regressions performed on sleep and sleep-related variables. The multiple regression coefficients were statistically significant for each dependent variable. The partial correlations indicate that:

**Table 4:** Multiple linear regression models with sleep and sleep-related measures as dependent variables and sociodemographic and clinical variables as predictors. Asterisks index statistical significance (*p* < 0.05)

Dependent variables	Predictors	β	t	*p*
**PSQI Global ***R* = 0.61; adjusted R^2^ = 0.37; F_17,708_ = 25.9; *p* < 0.001*	Diet (omniv/veg)	0.06	0.86	0.39
	Age	0.21	6.02	< 0.001*
	Gender (M/F)	-0.04	-0.58	0.56
	BMI	-0.002	-0.09	0.93
	Physical activity (Yes/No)	-0.09	-1.19	0.23
	Illness (Yes/No)	0.01	0.16	0.87
	Smoke (Yes/No)	0.02	0.34	0.73
	Alcohol			
	*Once a month or less vs. Never*	-0.007	-0.08	0.94
	*2/4 times a month vs. Never*	-0.13	-1.41	0.16
	*2/3times a week vs. Never*	-0.01	-0.09	0.92
	*≥ 4 times a week vs. Never*	0.23	1.49	0.13
	EDE	0.22	6.08	< 0.001*
	DASS-21	0.15	3.60	< 0.001*
	WHO-5	-0.33	-8.37	< 0.001*
	MEDAS	-0.02	-0.62	0.53
	rMEQ			
	*Evening - vs. Neutral type*	0.30	3.07	0.002*
	*Morning vs. Neutral type*	-0.15	-2.09	0.04*
**ISI***R* = 0.57; adjusted R^2^ = 0.31; F_17,708_ = 20.5; *p* < 0.001*	Diet (omniv/veg)	0.05	0.66	0.51
	Age	0.15	4.18	< 0.001*
	Gender (M/F)	-0.13	-1.71	0.09
	BMI	-0.05	-1.32	0.19
	Physical activity (Yes/No)	0.03	0.33	0.74
	Illness (Yes/No)	0.06	0.81	0.42
	Smoke (Yes/No)	-0.004	-0.06	0.96
	Alcohol			
	*Once a month or less vs. Never*	-0.03	-0.28	0.78
	*2/4 times a month vs. Never*	-0.15	-1.65	0.10
	*2/3times a week vs. Never*	0.004	0.03	0.97
	*≥ 4 times a week vs. Never*	0.37	2.25	0.02*
	EDE	0.20	5.47	< 0.001*
	DASS-21	0.20	4.51	< 0.001*
	WHO-5	-0.27	-6.61	< 0.001*
	MEDAS	-0.0005	-0.01	0.99
	rMEQ			
	*Evening - vs. Neutral type*	0.21	2.08	0.04*
	*Morning vs. Neutral type*	-0.15	-2.00	0.046*
**ESS***R* = 0.27; adjusted R^2^ = 0.05; F_17,708_ = 3.35; *p* < 0.001*	Diet (omniv/veg)	-0.08	-0.89	0.38
	Age	0.006	0.14	0.89
	Gender (M/F)	0.14	1.57	0.12
	BMI	0.02	0.48	0.63
	Physical activity (Yes/No)	-0.14	-1.52	0.13
	Illness (Yes/No)	0.04	0.43	0.67
	Smoke (Yes/No)	0.14	1.66	0.10
	Alcohol			
	*Once a month or less vs. Never*	0.10	0.82	0.41
	*2/4 times a month vs. Never*	-0.03	-0.31	0.76
	*2/3times a week vs. Never*	0.03	0.20	0.84
	*≥ 4 times a week vs. Never*	0.07	0.37	0.71
	EDE	0.09	2.08	0.04*
	DASS-21	0.15	2.87	0.004*
	WHO-5	-0.17	-0.36	0.72
	MEDAS	0.06	1.36	0.18
	rMEQ			
	*Evening - vs. Neutral type*	0.0009	0.007	0.99
	*Morning vs. Neutral type*	0.06	0.70	0.48
**STOP-Bang***R* = 0.75; adjusted R^2^ = 0.55; F_17,708_ = 53.4; *p* < 0.001*	Diet (omniv/veg)	-0.22	-3.53	< 0.001*
	Age	0.44	15.04	< 0.001*
	Gender (M/F)	-1.04	-17.15	< 0.001*
	BMI	0.15	5.54	< 0.001*
	Physical activity (Yes/No)	-0.08	-1.36	0.17
	Illness (Yes/No)	0.15	2.51	0.01*
	Smoke (Yes/No)	0.04	0.68	0.49
	Alcohol			
	*Once a month or less vs. Never*	0.04	0.52	0.60
	*2/4 times a month vs. Never*	-0.07	-0.95	0.34
	*2/3times a week vs. Never*	-0.06	-0.63	0.53
	*≥ 4 times a week vs. Never*	0.23	1.73	0.08
	EDE	0.21	7.11	< 0.001*
	DASS-21	0.08	2.35	0.02*
	WHO-5	-0.03	-0.79	0.43
	MEDAS	-0.04	-1.29	0.20
	rMEQ			
	*Evening - vs. Neutral type*	0.15	1.80	0.07
	*Morning vs. Neutral type*	0.06	1.01	0.31

1. Age, EDE, DASS-21, WHO-5, and rMEQ were predictors of the PSQI score. Poorer sleep quality was associated with older age, greater eating disorder psychopathology, higher depression, anxiety, and stress symptoms, lower psychological wellbeing, greater tendency to eveningness, and reduced tendency to morningness compared to neutral chronotype;
2. Age, Alcohol consumption, EDE, DASS-21 WHO-5, and rMEQ were predictors of the ISI score. Higher insomnia symptoms were associated with older age, alcohol use ≥ 4 times a week compared to no alcohol use, greater eating disorder psychopathology, higher depression, anxiety, and stress symptoms, lower psychological wellbeing, greater tendency to eveningness and reduced tendency to morningness compared to neutral chronotype;
3. EDE and DASS-21 were predictors of the ESS score: higher sleepiness was associated with greater eating disorder psychopathology and higher depression, anxiety, and stress symptoms;
4. Diet, age, gender, BMI, illness, EDE, and DASS-21 were predictors of the STOP-Bang score. A greater OSA risk was associated with omniv diet, older age, male gender, higher BMI, presence of illness, greater eating disorder psychopathology and higher depression, anxiety, and stress symptoms.

Table 5 reports the results of the multiple ordinal regressions performed on MUPS altered motor nocturnal behaviors. The regression coefficients were statistically significant for each dependent variable. The partial correlations indicate that:

**Table 5:** Multiple ordinal regression models with movement sleep variables assessed by the MUPS as dependent variables and sociodemographic and clinical variables as predictors. Asterisks index statistical significance (*p* < 0.05)

Dependent variables	Predictors	Odd Ratio	*p*	95% Confidence interval	
				Lower Bound	Upper Bound
**Hypnic Jerks** Negelkerke R^2^ = 0.06; χ_17_ = 114; *p* < 0.001*	Diet (omniv/veg)	1.54	0.008*	0.11	0.75
	Age	0.98	0.006*	-0.03	-0.004
	Gender (M/F)	1.25	0.18	-0.10	0.56
	BMI	0.99	0.39	-0.04	0.01
	Physical activity (Yes/No)	0.73	0.06	-0.64	0.01
	Illness (Yes/No)	1.49	0.01*	0.09	0.71
	Smoke (Yes/No)	1.08	0.64	-0.23	0.38
	Alcohol				
	*Once a month or less vs. Never*	2.03	0.001*	0.28	1.14
	*2/4 times a month vs. Never*	2.03	< 0.001*	0.30	1.12
	*2/3times a week vs. Never*	2.40	< 0.001*	0.36	1.39
	*≥ 4 times a week vs. Never*	3.30	< 0.001*	0.48	1.91
	EDE	1.14	0.03*	0.01	0.24
	DASS-21	1.03	< 0.001*	0.01	0.05
	WHO-5	0.99	0.68	-0.05	0.03
	MEDAS	1.08	0.04*	0.004	0.15
	rMEQ				
	*Evening - vs. Neutral type*	0.62	0.04*	-0.91	-0.03
	*Morning vs. Neutral type*	0.83	0.26	-0.51	0.14
**Rhythmic Foot Tremors** Negelkerke R^2^ = 0.04; χ^2^_17_ = 58.7; *p* < 0.001*	Diet (omniv/veg)	1.32	0.13	-0.08	0.64
	Age	1.00	0.72	-0.01	0.01
	Gender (M/F)	0.86	0.43	-0.51	0.22
	BMI	1.01	0.62	-0.02	0.04
	Physical activity (Yes/No)	1.14	0.49	-0.24	0.51
	Illness (Yes/No)	1.27	0.18	-0.11	0.59
	Smoke (Yes/No)	1.37	0.07	-0.02	0.65
	Alcohol				
	*Once a month or less vs. Never*	1.20	0.46	-0.31	0.69
	*2/4 times a month vs. Never*	1.22	0.42	-0.27	0.68
	*2/3times a week vs. Never*	1.48	0.17	-0.17	0.97
	*≥ 4 times a week vs. Never*	1.37	0.44	-0.50	1.11
	EDE	0.98	0.72	-0.15	0.10
	DASS-21	1.06	< 0.001*	0.04	0.07
	WHO-5	1.04	0.07	-0.004	0.09
	MEDAS	0.97	0.56	-0.11	0.06
	rMEQ				
	*Evening - vs. Neutral type*	1.01	0.97	-0.49	0.50
	*Morning vs. Neutral type*	1.08	0.68	-0.29	0.44
**Rhythmic Movement Disorder** Negelkerke R^2^ = 0.05; χ^2^_17_ = 43.5; *p* < 0.001*	Diet (omniv/veg)	1.20	0.50	-0.35	0.70
	Age	0.99	0.43	-0.02	0.01
	Gender (M/F)	1.01	0.97	-0.52	0.56
	BMI	1.00	0.99	-0.06	0.04
	Physical activity (Yes/No)	1.24	0.44	-0.32	0.78
	Illness (Yes/No)	0.70	0.20	-0.93	0.18
	Smoke (Yes/No)	1.62	0.05	-0.007	0.96
	Alcohol				
	*Once a month or less vs. Never*	1.13	0.75	-0.60	0.87
	*2/4 times a month vs. Never*	0.94	0.86	-0.76	0.67
	*2/3times a week vs. Never*	0.81	0.62	-1.09	0.65
	*≥ 4 times a week vs. Never*	2.92	0.03*	0.09	2.04
	EDE	1.01	0.89	-0.17	0.19
	DASS-21	1.05	< 0.001*	0.02	0.08
	WHO-5	1.06	0.08	-0.007	0.12
	MEDAS	0.99	0.82	-0.14	0.11
	rMEQ				
	*Evening - vs. Neutral type*	1.66	0.11	-0.13	1.12
	*Morning vs. Neutral type*	1.05	0.86	-0.51	0.60
**Periodic Leg Movements** Negelkerke R^2^ = 0.03; χ^2^_17_ = 33.9; *p* = 0.009*	Diet (omniv/veg)	1.05	0.84	-0.41	0.49
	Age	0.98	0.06	-0.03	0.0004
	Gender (M/F)	0.74	0.18	-0.72	0.14
	BMI	1.01	0.67	-0.03	0.04
	Physical activity (Yes/No)	1.08	0.74	-0.37	0.54
	Illness (Yes/No)	1.02	0.94	-0.43	0.45
	Smoke (Yes/No)	1.12	0.60	-0.31	0.52
	Alcohol				
	*Once a month or less vs. Never*	1.35	0.36	-0.34	0.97
	*2/4 times a month vs. Never*	1.27	0.45	-0.36	0.88
	*2/3times a week vs. Never*	1.92	0.07	-0.05	1.38
	*≥ 4 times a week vs. Never*	2.78	0.03*	0.05	1.96
	EDE	1.06	0.41	-0.09	0.21
	DASS-21	1.02	0.09	-0.0003	0.04
	WHO-5	0.99	0.61	-0.07	0.04
	MEDAS	1.03	0.51	-0.07	0.14
	rMEQ				
	*Evening - vs. Neutral type*	0.73	0.30	-0.92	0.26
	*Morning vs. Neutral type*	0.73	0.20	-0.79	0.15
**Nocturnal Leg Cramps** Negelkerke R^2^ = 0.04; χ^2^_17_ = 64.1;*p* < 0.001*	Diet (omniv/veg)	1.08	0.64	-0.26	0.42
	Age	1.03	< 0.001*	0.02	0.04
	Gender (M/F)	1.31	0.12	-0.07	0.61
	BMI	0.99	0.69	-0.03	0.02
	Physical activity (Yes/No)	0.71	0.05	-0.68	0.001
	Illness (Yes/No)	0.97	0.86	-0.36	0.30
	Smoke (Yes/No)	1.25	0.18	-0.11	0.56
	Alcohol				
	*Once a month or less vs. Never*	0.10	0.99	-0.45	0.44
	*2/4 times a month vs. Never*	0.97	0.90	-0.45	0.39
	*2/3times a week vs. Never*	0.73	0.24	-0.85	0.21
	*≥ 4 times a week vs. Never*	1.08	0.83	-0.63	0.77
	EDE	1.07	0.27	-0.05	0.18
	DASS-21	1.03	0.004*	0.009	0.04
	WHO-5	0.98	0.48	-0.06	0.03
	MEDAS	0.96	0.26	-0.13	0.03
	rMEQ				
	*Evening - vs. Neutral type*	0.67	0.10	-0.89	0.07
	*Morning vs. Neutral type*	1.06	0.72	-0.28	0.39

5. Diet, age, illness, alcohol consumption, EDE, DASS-21, MEDAS, and rMEQ were predictors of hypnic jerks. Veg diet, younger age, presence of illness, every alcohol consumption frequency compared to no alcohol use, greater eating disorder psychopathology, stronger adherence to the Mediterranean diet, and reduced tendency toward eveningness compared to neutral chronotype were associated with higher hypnic jerks frequency;
6. DASS-21 was a predictor of rhythmic foot tremors: greater depression, anxiety, and stress symptoms were associated with more frequent rhythmic foot tremors;
7. Alcohol consumption and DASS-21 were predictors of rhythmic movement disorder. Greater depression, anxiety, and stress symptoms and alcohol use ≥ 4 times a week compared to no alcohol use were associated with higher rhythmic movement disorder frequency;
8. Alcohol consumption was a predictor of periodic leg movements: alcohol use ≥ 4 times compared to no alcohol use was associated with more frequent periodic leg movements;
9. Age and DASS-21 were predictors of nocturnal leg cramps. Older age and greater depression, anxiety, and stress symptoms were associated with higher nocturnal leg cramps frequency.

## Discussion

The aim of the study was to assess the relationship between sleep variables and veg diets in an Italian sample considering the role of several factors. The main findings show that: (a) compared to ominv, veg exhibited a lower risk of OSA and a higher hypnic jerks frequency; (b) considering the role of several variables, the dietary pattern only predicted OSA risk and hypnic jerks. Notably, omniv individuals showed a greater tendency to evening chronotype than veg participants.

Since the study was aimed to clarify the relationship between dietary patterns and sleep-related variables, the discussion will be focused on this topic.

Veg diet was associated with reduced OSA risk, supporting the relevance of diet in OSA patients (e.g., [30]). Melaku and coworkers [31] observed in a USA sample that OSA was inversely related to adherence to plant-based, healthy plant-based, and pro-veg diet index, and positively related to unhealthy plant-based diet index, also considering sociodemographic variables, behavioural factors, and chronic conditions. We extended this finding to an Italian sample, adding the direct comparison between veg and omniv and considering the influence of other variables.

A possible explanation for this relationship is that veg diets reduce the risk of obesity, one of the main predictors of OSA. Consistently, we found a lower BMI in veg compared to omniv, and greater BMI and higher eating disorder symptomatology also predicted increased OSA risk. Veg diets have a protective role against obesity [2] and promote an anti-inflammatory effect [32]. Recent findings suggest that the relationship between lifestyle, diet, and OSA may be mediated by the interaction between the status of chronic conditions, systemic inflammation, adiposity, and muscle mass/tone [30]. Therefore, it is possible that veg diets may reduce the risk of OSA by a positive effect on obesity and inflammation.

Veg diets are generally more frequent among young women e.g., [33, 34] while OSA risk is greater in postmenopausal women due to hormonal factors and chronological aging [35]. In this view, it is difficult to determine a possible impact of the reduced number of veg women with age and the increased OSA risk, since hormonal changes and chronological aging represent relevant confounding factors. In the current study, this issue has been only statistically controlled by including sex and age in our multiple regression models. Results show that the role of diet as a predictor of OSA risk remains significant also considering these factors. However, we emphasize the need to disentangle the specific roles and interactions of diets, hormones, and chronological aging in OSA risk in women.

The relationship between hypnic jerks and veg diet represents an original finding. Hypnic jerks are benign phenomena characterized by sudden non-periodic myoclonic movements during the sleep onset [36]. Although hypnic jerks are prevalent in the healthy population and can be triggered by several factors [37], their excessive presence has been described in several neurological disorders and should be treated in case of significant interference with sleep and life quality. Several hypotheses about their origin [38] point to subcortical generators, altered sensory processing, and response to hypnagogic imagery. However, different physiological types of motor patterns have been described [39], making their pathophysiology uncertain.

To the best of our knowledge, the relationship between hypnic jerks and diet has never been systematically explored. Considering that a particularly restrictive veg diet may induce a deficiency of vitamins and minerals [2], it is possible that a paucity in nutrients that affect neuromuscular function and sleep regulation may be associated with increased hypnic jerk’s frequency. In particular, the deficiency of iron, relevant for regulation of dopamine synthesis and receptors, has been considered a factor involved in the pathophysiology of sleep-related movement disorders [16]. A deficiency in nutrients like iron, magnesium or calcium could induce involuntary muscle movements, potentially including hypnic jerks. A low level of magnesium can impact on the higher hypnic jerk frequency in veg due to the muscle relaxant properties of this micronutrient [40], relevant for the sleep onset process [41]. It is worth noting that also low levels of B-Vitamins may affect the sleep onset process [42], and some evidence suggest that they can protect against muscle cramps in selected populations e.g., [43, 44]. On the other hand, a psychological factor may act as a confounding factor in the relationship between diet and hypnic jerks. Indeed, a greater sensitivity to sleep health may imply a better identification of hypnic jerks as sleep problems. Veg individuals are often motivated by health aspects when starting their plant-based diet and frequently engage in healthier lifestyle habits [45, 46]. In this view, participants in the veg sample, following health-promoting behaviours more likely than omniv, may be more sensitive to sleep issues, leading to an over-reporting of sleep onset jerks. The fact that our veg sample had a greater proportion of women may also impact on this point, since women often exhibit a greater subjective sensitivity to sleep issues, driven by neurobiological, hormonal, and psychosocial factors [47, 48]. However, other sleep variables on which a greater attention to health issues may have an impact, like self-reported sleep quality, did not showed a relationship with the dietary habits in our study. Although the available literature does not report a gender difference in self-reported hypnic jerks, this potential confound should be directly investigated.

Concerning the absence of a relationship between dietary patterns and other sleep-related movement disorders, the frequency of the movement disorders should be considered before reaching definitive conclusions. Indeed, rhythmic movement disorder and periodic leg movements showed a very low frequency, making it difficult to provide definitive conclusions about their relationship with diet. A specific assessment of the dietary pattern in selected clinical populations affected by sleep-related movement disorders is needed.

Although we used chronotype as a predictor of sleep-related measures, the observed greater tendency to eveningness in omniv compared to veg represents an interesting finding. Evening chronotype is associated with more frequent engagement in unhealthy dietary habits [19], obesity [49], and lower adherence to the Mediterranean diet [50]. A recent study found an association between later chronotype and unhealthy plant-based diet quality, while a healthy pant-based dietary index was related to an earlier chronotype [51]. Our result of lower tendency to eveningness in veg participants is consistent with these findings. Since individuals with evening chronotypes are more likely to exhibit poorer mental health [52], the possibility of considering the dietary pattern as an additional target to improve wellbeing in individuals with late chronotype should be assessed.

In the present study, the dietary pattern was not associated with sleep quality, insomnia symptoms, and sleepiness. The literature on these sleep-related variables in completely veg diets is small and heterogenous. Our findings align with those studies showing the absence of a relationship of veg diets with sleep quality [9, 10] and insomnia symptoms [10, 11]. Only one study previously assessed sleepiness in veg subjects, showing that these participants were less sleepy than omniv ones [6], but this study was performed during the COVID-19 pandemic, reporting only prevalence analyses. Overall, our findings suggest that when relevant variables for sleep and nutrition are considered, no relationship can be observed between dietary pattern and self-reported sleep quality, insomnia, and sleepiness. In this view, the positive effect of a veg diet on these variables observed in previous studies may be indirect. On the other hand, albeit we controlled for the absence of differences between vegans and vegetarians in sleep variables, we did not distinguish different types of veg diets or defined healthy and unhealthy veg dietary patterns. Unhealthy plant-based diets negatively affect poor sleep quality and duration. Future studies should clarify the impact of specific veg diets and their healthy/unhealthy pattern on sleep.

Some limitations should be considered. The online-based recruitment strategy limits our experimental control on the sample and may introduce relevant biases, also resulting in an unbalanced sample concerning several sociodemographic variables. Moreover, while the larger part of the omniv sample had always followed this dietary pattern, the interval between the beginning of the present dietary pattern and the data collection was more variable across veg participants. As previously discussed, veg dietary patterns are heterogeneous, and healthy and unhealthy veg diets may have the opposite effect on sleep variables. This potential heterogeneity may have masked some relationships between the diet and sleep variables, and several conclusions remain speculative. Also, we did not consider herbal drinks and supplements as possible confounding factors. Finally, although we included an assessment of the adherence to the Mediterranean diet beyond the self-definition of the dietary pattern, in the present study we cannot completely account for the fact that several self-identified veg individuals actually consume meat or fish [53–55]. These individuals do not strictly adhere to an operational definition of vegetarianism but report themselves as veg and cannot be detected by our online survey, limiting the generalizability of our findings. This tendency may be influenced by self-perception of the dietary choices and motivation in the self-defining as veg [55]. In particular, social desirability has been previously associated with the tendency to over-report foods perceived as healthy and under-report less healthy foods [56], particularly in females [57]. Future studies on the relationship between self-reported dietary pattern and sleep should control for social desirability as a potential confounding factor, and consumer studies focused on the food shopped/consumed are needed in this field to discriminate “actual” veg from veg-inclined individuals while assessing sleep features.

## Conclusion

The relationship between sleep and nutrition is multifaced and influenced by several factors. Sleep represents a complex phenomenon, and the idea that something just as complex as a specific dietary pattern influences sleep variables in a single direction would be simplistic. Our findings suggest that the main positive effect on sleep of a veg diet compared to an omniv one is represented by reduced OSA risk. However, the increased frequency of hypnic jerks in veg diets should be underlined: albeit it does not represent a pathological condition per se, it may indicate a deficiency in nutrients relevant for neuromuscular functioning with possible detrimental consequences on health and sleep. Finally, we did not observe an influence of veg diet on sleep quality, insomnia symptoms, and sleepiness, suggesting that a veg diet per se does not directly affect these sleep variables.

Future studies should be aimed at understanding the specific effect on sleep variables of distinct veg dietary patterns. Also, it should be clarified which factors and in which specific populations the adherence to a veg diet can have an impact that, in turn, may affect specific sleep variables. Finally, a stronger research effort is needed to assess the influence of veg diets on physiological sleep measures.

## Supplementary Information

Below is the link to the electronic supplementary material.

Supplementary Material 1 (DOCX 17.9 KB)

## Data Availability

Data will be made available upon reasonable request.
