# Supplementary material for: Sleeping green: an Italian survey for the assessment of the relationship between sleep and vegetarian diet
Source: Sleep Breath. 2026 Mar 10;30(1):85. doi: 10.1007/s11325-026-03593-3 (PMC12975816; doi:10.1007/s11325-026-03593-3)
Supplement: Supplementary file 1 — Supplementary Material 1 (DOCX 17.9 KB) [file 11325_2026_3593_MOESM1_ESM.docx]

**Sleeping green: an Italian survey for the assessment of the relationship between sleep and vegetarian diet**

**SUPPLEMENTARY MATERIAL**

**Excluded cases**

We received 781 questionnaires. One individual was excluded because he/she did not provide the informed consent, and one because reported age <18 y. Three individuals completed the survey more than once: in these cases, only the first one was considered. Since pescetarians were only 25, and individuals that responded “other” to the question about gender were only 4, these groups were excluded from the analyses. Our final sample included 747 participants: 532 omniv and 215 veg.

**Diet duration**

Our final sample included 747 participants: 532 omniv and 215 veg. Among the omniv, 2 (0.37%) participants followed an omniv diet for less than one year, 8 (1.50%) between one and five years, 8 (1.50%) from more than 5 years, and 514 (96.62%) have always followed an omniv diet. Among the veg, 19 (8.84%) participants followed a veg diet for less than one year, 92 (42.79%) between one and five years, 99 (46.05%) for more than 5 years, and 5 (2.33%) have always followed a veg diet.

**Comparison between vegan and vegetarians on sleep-related measures**

Sleep and sleep-related variables (PSQI global score, ESS, STOP-Bang, ISI) were compared between vegan and vegetarians by unpaired Student’s t-tests. PSQI components and MUPS sleep-related movement disorders were compared between vegan and vegetarians and veg by Mann-Whitney U. Table S1 reports the results of these comparisons, showing the absence of significant differences between vegans and vegetarians in sleep and sleep-related measures.

**Table S1.** Sleep and sleep-related variables in the total sample and in vegan and vegetarian groups. The comparisons between groups have been performed using unpaired Student’s t-tests for PSQI Global, ISI, ESS, and STOP-Bang, and using Mann-Whitney U for PSQI components and MUPS sleep-related movement disorders. The alpha level was set at 0.05. The asterisk indexes a significant difference.

|  | **Vegan**  **(N=118)** | **Vegetarian**  **(n=97)** | **Vegan vs. Vegetarian** |
| --- | --- | --- | --- |
| **Sleep and sleep-related variables** | **Mean (SD)** | | ***t* (*p*)** |
| ESS | 5.34 (3.62) | 4.91 (3.47) | 0.90 (0.36) |
| STOP-Bang | 1.02 (1.09) | 0.95 (1.17) | 0.44 (0.66) |
| ISI | 6.34 (5.44) | 6.40 (5.25) | -0.09 (0.93) |
| PSQI Global | 6.19 (3.53) | 5.77 (3.20) | 0.89 (0.37) |
| *PSQI Components* | **Mean (SD)** | | ***p*** |
| C1 – Subjective sleep quality | 1.16 (0.71) | 1.05 (0.70) | 0.36 |
| C2 – Sleep latency | 0.90 (0.91) | 0.98 (0.85) | 0.39 |
| C3 – Sleep duration | 1.11 (0.91) | 0.92 (0.85) | 0.14 |
| C4 – Sleep efficiency | 0.69 (0.94) | 0.60 (0.95) | 0.29 |
| C5 – Sleep disturbance | 1.10 (0.48) | 1.06 (0.50) | 0.45 |
| C6 – Use of sleep medications | 0.27 (0.79) | 0.13 (0.45) | 0.60 |
| C7 – Daytime dysfunction | 0.96 (0.78) | 1.03 (0.74) | 0.39 |
| **Sleep-related movement disorders** | **Mean (SD)** | | ***p*** |
| Hypnic Jerks | 2.60 (1.79) | 2.76 (1.77) | 0.47 |
| Rhythmic Foot Tremors | 1.23 (1.86) | 1.61 (1.96) | 0.12 |
| Rhythmic Movement Disorder | 0.47 (1.23) | 0.37 (1.15) | 0.51 |
| Periodic Leg Movements | 0.49 (1.10) | 0.46 (1.12) | 0.57 |
| Nocturnal Leg Cramps | 1.22 (1.51) | 1.31 (1.54) | 0.68 |
